# Nonadherence to daily self-weighing and activity tracking is associated with weight fluctuations among African American breast cancer survivors

**DOI:** 10.1371/journal.pone.0199751

**Published:** 2018-06-26

**Authors:** Chantel L. Martin, Deborah F. Tate, Carmina G. Valle

**Affiliations:** 1 Department of Health Behavior, Gillings School of Global Public Health, University of North Carolina, Chapel Hill, NC, United States of America; 2 Department of Nutrition, Gillings School of Global Public Health, University of North Carolina, Chapel Hill, NC, United States of America; 3 Lineberger Comprehensive Cancer Center, University of North Carolina, Chapel Hill, NC, United States of America; Vanderbilt University, UNITED STATES

## Abstract

**Introduction:**

Daily self-weighing (DSW) and daily activity tracking (DAT) are useful strategies for preventing weight gain among African American breast cancer survivors. However, self-monitoring behaviors vary over time, increasing risk of weight gain. This study explored the association of nonadherence to DSW and DAT with corresponding weight fluctuations among African American breast cancer survivors.

**Methods:**

Using data from a 6-month randomized controlled trial, we conducted a secondary data analysis among women randomized into a DSW group (n = 13) and a DSW+DAT group (n = 11). DSW and DAT were captured from wireless scale and activity tracker data. Nonadherence to DSW was defined as one or more days without a weight measurement, and nonadherence to DAT was defined as one or more days without activity tracking. Generalized estimating equations were used to examine weight fluctuations in relation to nonadherence to DSW and DAT. Data analysis occurred from September 2016-April 2017.

**Results:**

Over the 6-month study period, women provided 119.2 ± 46.0 weight measurements and 121.9 ± 53.2 days of physical activity tracking. Nonadherence to DSW was associated with weight fluctuations. For every 1-day increase in nonadherence to DSW, weight increased by 0.031 kg (95% CI: 0.012, 0.050; *p*<0.01). Additionally, during periods of DSW and DAT weight decreased by 0.028 kg (95% CI: -0.042, -0.014; *p*<0.001) and 0.017 kg (95% CI: -0.030; -0.004) respectively.

**Conclusions:**

Our findings suggest that nonadherence to DSW was associated with weight gain among breast cancer survivors. Weight loss was enhanced during periods of DSW and DAT.

## Introduction

Breast cancer is the most commonly diagnosed cancer among women [1]. African American breast cancer survivors have higher rates of cancer-related morbidities [2, 3], are more likely to experience obesity [4], and may be at increased risk for post-diagnosis weight gain than other women [5]. Therefore, weight gain prevention has important public health implications for improving quality of life and survival among a high-risk population of breast cancer survivors.

Self-monitoring is the foundation of behavioral weight control programs. Rooted in self-regulation theory, self-monitoring of diet, physical activity, and/or weight provides individuals feedback to heighten self-awareness of progress towards a goal and how behaviors are impacting goal achievement [6, 7]. Frequent self-monitoring of body weight and exercise is linked to greater weight loss and weight maintenance success and is a recommended component of standard behavioral treatment for weight loss [6, 8, 9]. Self-monitoring of weight and activity on a regular basis provides opportunities for individuals to improve self-awareness, obtain feedback and reinforcement, detect weight fluctuations, and facilitates making changes in diet and exercise behaviors based on frequent weight information [10]. However, self-monitoring tends to decline over time, as careful monitoring of dietary intake and physical activity behaviors can be time-intensive; therefore, daily self-weighing (DSW) and activity tracking (DAT) has emerged as a lower-intensity and potentially more sustainable approach to self-monitoring.

Evidence from weight management programs generally supports daily weighing as the recommended self-weighing frequency. Previous interventions that have encouraged DSW have shown that individuals who weigh daily are more likely to prevent weight gain than those who weigh less frequently [11–14]. The effectiveness of DSW may be a result of adoption of weight control behaviors (e.g., diet and physical activity), as more frequent self-weighing has been shown to be associated with less fat intake, more walking, increases in self-monitoring of intake, and greater dietary restraint [7, 11, 15, 16]. Moreover, individuals who are encouraged to track physical activity daily are more likely to manage their weight [6, 17–19].

Technology-based instruments, such as wireless scales and wearable activity trackers, offer a unique opportunity for weight control interventions to encourage daily self-monitoring and provide real-time feedback based on continuously monitored weight and activity data. In a 6-month randomized trial among overweight adults that included cellular-connected smart scales for daily self-monitoring of weight, the intervention group lost significantly more weight than the control group [20]. Additionally, Martin et al. evaluated a 12-week smartphone-based weight loss intervention for overweight and obese adults using a wireless scale and activity tracker for daily self-monitoring of weight and exercise and found that participants in the intervention group achieved significantly greater weight loss than the health education control group [21].

Continuously monitored objective data from these weight control interventions enable the estimation of temporal variations in self-monitoring frequency. Two recent studies investigated the impact of changes in self-monitoring of weight and activity in relation to weight fluctuations, finding that periods of nonadherence to DSW and daily activity tracking (DAT) were associated with weight gain [22, 23]. Further, data from a three-arm 6-month pilot intervention trial found that DSW and DAT, as part of a technology-delivered behavioral intervention, were promising for preventing weight gain among African American breast cancer survivors [17].

Given that self-monitoring behaviors vary over time, identifying periods of nonadherence when breast cancer survivors may be at risk of gaining weight could inform the development of future weight control interventions to help reduce morbidity and mortality in this population. The purpose of this paper was to examine the association of nonadherence to DSW and DAT with corresponding weight fluctuations using data from African American breast cancer survivors who participated in a 6-month weight gain prevention intervention[17]. In a previous pilot study, we tested the feasibility and preliminary efficacy of two remotely delivered interventions that encouraged DSW (+ DAT) and utilized objective monitoring and tailored feedback to promote self-regulation for weight gain prevention. Intervention participants who were encouraged to self-monitor both weight and physical activity daily achieved a median weight change of -0.9% at 6 months compared to a 0.2% gain in the control group. We hypothesized that during periods of nonadherence to DSW and DAT, women participating in the intervention would experience weight gain.

## Materials and methods

### Study participants

Data are from a randomized controlled pilot trial of two 6-month self-regulation interventions for weight gain prevention among African American breast cancer survivors [17]. Eligibility criteria for women to participate included: being female; aged 18 years or older; self-identified as African American or black; body mass index (BMI) of 20–45 kg/m^2^; ability to read, write, and speak English; having regular access to the Internet and computer; use email; diagnosed with stage I-IIIA breast cancer within the last 10 years; completed cancer treatment (except endocrine treatment); no evidence of progressive disease or second primary cancer; physician’s consent for participation; and willingness to be randomized. Participants were recruited over a 9-month period using a hospital-based health registry/cancer survivorship cohort, clinic-based in-person recruitment, direct mailings, community events, flyers, social media, and email. Participants (n = 35) were randomized over a 9-month period in 2014 to one of three groups: (1) a DSW intervention (INT; n = 13); (2) a DSW+DAT intervention (INT+; n = 11), or (3) a delayed-intervention control (n = 11). This analysis only includes women randomized to the intervention groups (n = 24), as control group participants were not encouraged to weigh themselves daily. Data collection occurred from January 2014 to June 2015 in Chapel Hill, NC. The Protocol Review Committee of the UNC Lineberger Comprehensive Cancer Center and the Institutional Review Board of the University of North Carolina at Chapel Hill reviewed and approved all study procedures.

### Study design

A description of the intervention was previously published [17]. Briefly, the aim of the 6-month intervention was weight gain prevention through self-regulation of eating and exercise behaviors. Participants in both intervention groups were encouraged to use DSW as their primary self-monitoring strategy and received a Bluetooth and Wi-Fi-enabled wireless scale (Withings WS-30, Cambridge, MA) [24] with access to a mobile app and website that displayed personalized weight trends, an individual face-to-face session, weekly email delivered behavioral lessons, and weekly emails with tailored feedback on self-weighing and weight data.

Participants in the INT+ group received all of the above in addition to being asked to wear an activity tracker (Withings Pulse, Cambridge, MA) [25]. This group was encouraged to track their daily activity and weigh themselves daily. Participants were encouraged to wear and monitor their physical activity daily using the activity trackers. Physical activity recommendations included a gradual increase of moderate-intensity exercise to 150–225 min/week (30–45 min/day on 5 days/week). The activity tracker synced data to a single online account that also interacted with the wireless scale. Tailored feedback encouraging and emphasizing daily activity tracking was provided to participants in this group based on both physical activity information from the activity tracker and weight data from the wireless scale.

### Measures

Participants were assessed at baseline, 3 months, and 6 months. Anthropometric data were collected during clinic visits by the study interventionist or other trained research staff members. Baseline measurements were used to determine starting weight for participants. Demographic data were collected via online qusetionnaires. Self-monitoring data were downloaded weekly by a research assistant and reviewed by the interventionist for accuracy. Two of five trained study staff double-entered all data into a database.

#### Demographics

Participants reported age, education, marital status, income, employment status, smoking behaviors, weight history, medication use, comorbidity and cancer history at baseline.

#### Anthropometric data

Baseline height was collected using a wall-mounted stadiometer by the interventionist or a research staff member. The average of two measures were used. Baseline weight was measured in light clothing, without shoes, using a calibrated digital scale (Tanita BWB-800). Two weight measurements were taken and averaged. Height and weight were used to calculate body mass index (BMI: kg/m^2^).

#### Adherence to self-monitoring

Wireless scales (Withings WS-30, Cambridge, MA) [24] were used to collect daily weights, which were transmitted directly to a companion app and website that was accessible through an online profile. Research assistants downloaded and recorded objective daily weight data for each participant. Weight data from the wireless scales were also used to calculate total number of days weighed and average number of days weighed per week. In the event of 2 or more weight measures in one day, we utilized the first recorded weight.

Self-monitoring of physical activity was objectively measured using activity trackers (Withings Pulse, Cambridge, MA) [25] for participants in the INT+ intervention group. Activity trackers transmitted data to the companion app and website, which was also accessible through the online profile. Daily activity data, including minutes of soft, moderate, and intense activity, as specified from the device (the equivalent to standard light, moderate, and vigorous classifications for physical activity), were downloaded and recorded weekly. Total number of days tracked, average number of days tracked per week, and a measure of tracking on 5 or more days per week were derived.

### Statistical analysis

Fisher’s exact tests for categorical variables and Kruskal-Wallis tests for continuous variables were used to compare differences in selected baseline characteristics between the INT and INT+ groups. For participants in both intervention groups (INT and INT+), nonadherence to DSW was defined as one or more days without a weight measurement (from wireless scales). Further, nonadherence to DAT was defined as one or more days with no activity tracking (i.e., no data or <1000 steps/day indicating limited wear time) among INT+ participants. The outcome variable of interest was weight fluctuations between two consecutive days of weight and activity measurements. Generalized estimating equations (GEE) models were used to examine the association between nonadherence to DSW and weight fluctuations (kg) among women in both intervention groups. GEE was also used to assess the association between nonadherence to DAT and weight fluctuations among women in the INT+ group. We chose to use GEE due to convergence issues encountered from a small sample when linear mixed models were employed. For DSW, the *β* coefficient from the GEE model represents weight change as a function of nonadherence to DSW (i.e., number of days between weight measurements), while the α coefficient corresponds to weight change as a function of adherence to DSW (i.e., when difference between days of weight measurements is zero). For DAT, the *β* coefficient from the GEE model represents weight change as a function of nonadherence to DAT (i.e., number of days between recorded physical activity data). The α coefficient corresponds to weight change as a function of adherence to DAT (i.e., when difference between days of tracked physical activity is zero). Models were examined with and without adjustment for baseline weight. Data analysis occurred from September 2016-April 2017. All statistical analyses were performed using SAS 9.4 (Cary, NC) with α<0.05 set *a priori* as the level of significance.

## Results

Participants were on average 52.4±8.2 years of age and obese (BMI: 33.3±5.7; **Table 1**). Women randomized to the INT+ group had a slightly higher BMI than women randomized into the INT group based on weight measured during the baseline clinic visit. Over the 6-month intervention period, women provided a total of 2834 self-monitored weight measurements from the wireless scales. Women in the INT+ group provided a higher number of weight measurements (135.6 ± 41.9) than the INT group (105.2 ± 46.2) over the possible 168 days during the study period. Specifically, women in the INT+ group self-monitored their weight on 81% of the days compared to 63% by the INT group. The 11 women randomized to the INT+ group recorded 1341 bouts of physical activity (121.9 ± 53.2 per woman; 73% of the 168 days of the study period).

**Table 1:** Selected baseline characteristics of WELL Body participants from two intervention groups (*n* = 24).

	Overall (*n* = 24)	INT^a^ (*n* = 13)	INT+^b^ (*n* = 11)	*p-*value^c^
Age, Mean ± SD	52.4 ± 8.2	52.6 ± 9.4	52.2 ± 6.9	1.00
Baseline BMI, Mean ± SD	33.3 ± 5.7	32.7 ± 6.1	34.0 ± 5.3	0.28
≤High school education level, *n* (%)	2 (8.3)	2 (15.4)	0 (0.0)	0.48
Income <$60,000 per year, *n* (%)	10 (41.7)	5 (50.0)	5 (50.0)	1.00
Married, *n* (%)	12 (50.0)	7 (53.8)	5 (45.5)	1.00
Premenopausal, *n* (%)	5 (20.8)	3 (23.1)	2 (18.2)	1.00
Number of weight measurements per woman, Mean ± SD	119.2 ± 46.0	105.2 ± 46.2	135.6 ± 41.9	0.06
Number of physical activity measurements per women, Mean ± SD	121.9 ± 53.2	---	121.9 ± 53.2	---
Number of weight measurements	2858	1366	1492	---
Number of days of physical activity	1341	---	1341	---

^a^*INT*, daily self-weighing intervention

^b^*INT+*, daily self-weighing + activity monitoring intervention

^c^*P*-values based on Kruskal-Wallis tests for continuous variables and Fisher’s exact tests for categorical variables.

**Fig 1:**
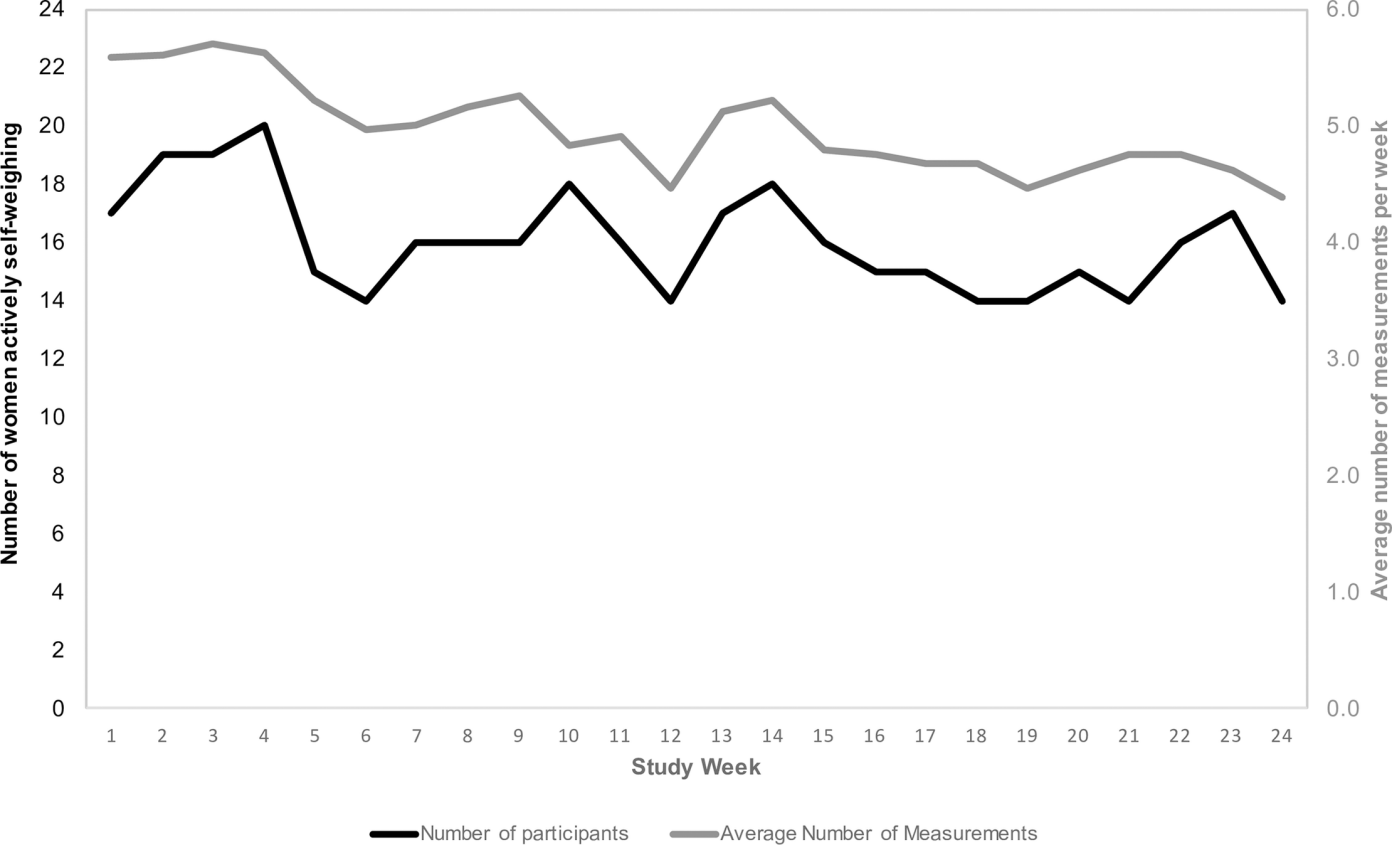
Patterns of self-weighing based on the number of women actively self-weighing. The left y-axis (and black line) represents the number of women actively self-weighing (5+ days per week). The right y-axis (and gray line) represents the average number of weight measurements per week.

**Fig 2:**
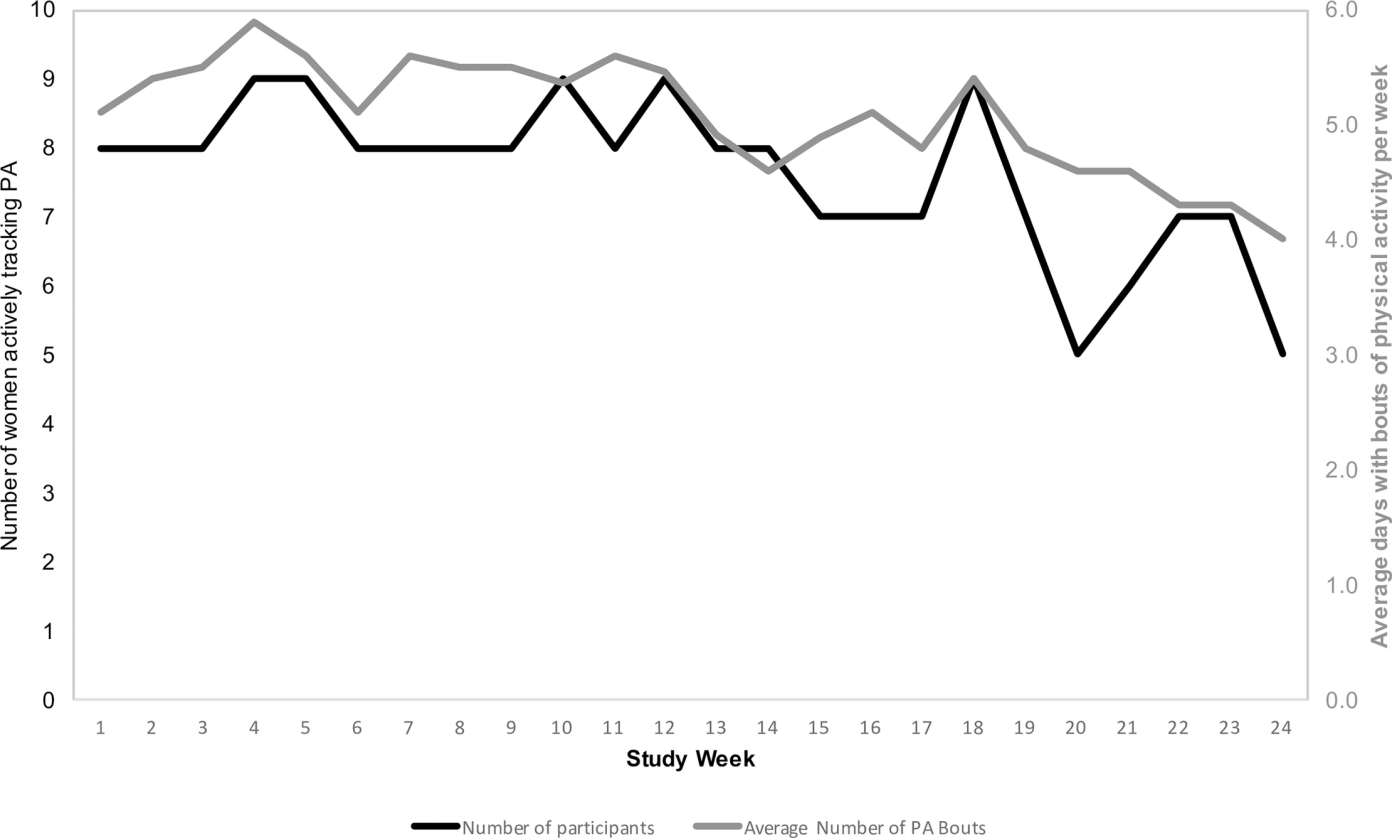
Patterns of activity tracking based on the number of women actively tracking physical activity. The left y-axis (and black line) represents the number of women actively tracking physical activity (5+ days per week). The right y-axis (and gray line) represents the average number of days per week of recorded of physical activity.

**Fig 3:**
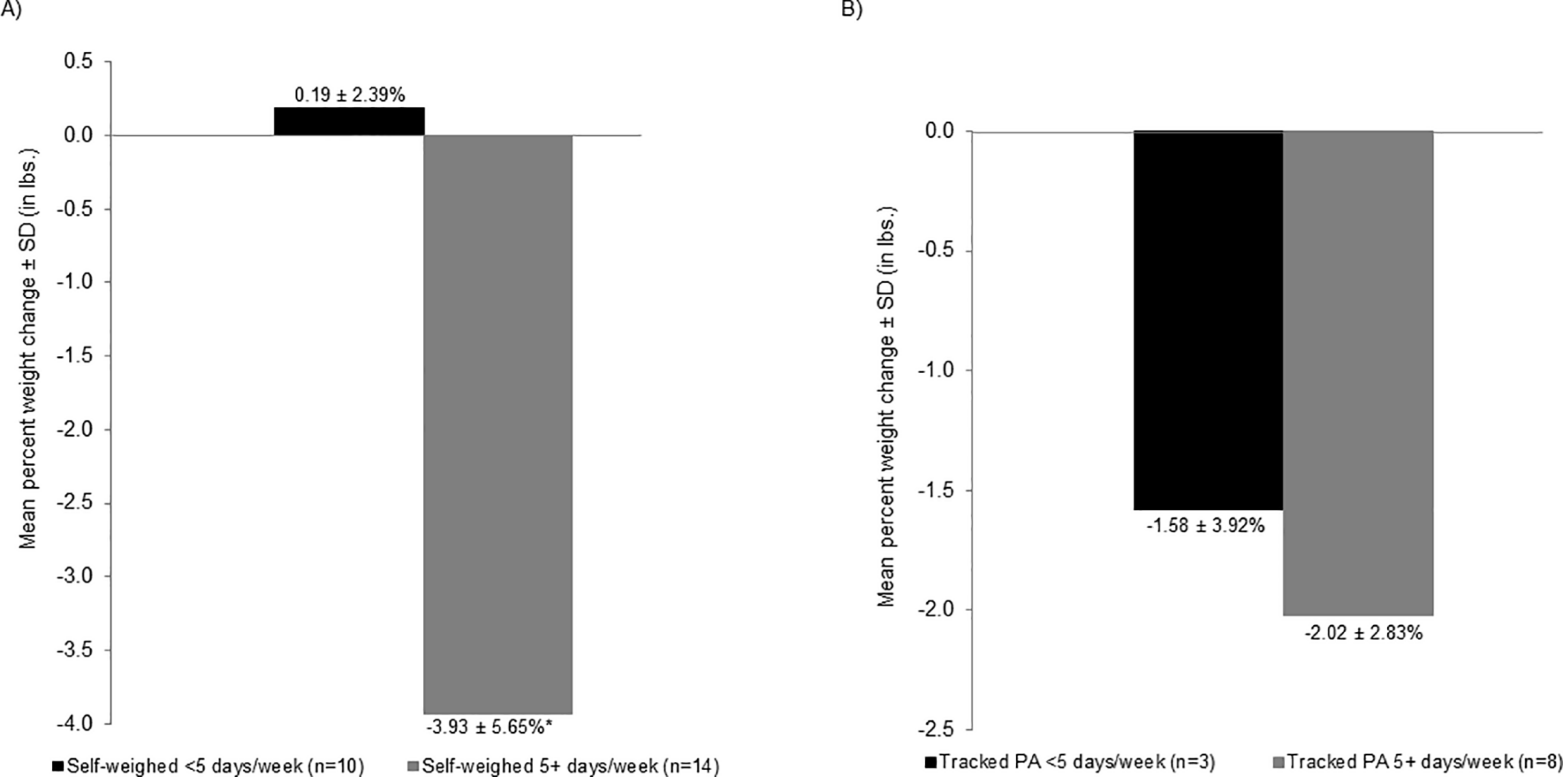
Mean percent weight change (in lbs.) over the 6-month study period. (A) self-weighing frequency and (B) PA tracking frequency. *P<0.05.

**Fig 1** shows weekly patterns of DSW over the study period. On average, women self-weighed 5.58 ± 1.79 days per week in week 1 compared to 4.38 ± 2.93 days per week in week 24 (p = 0.09). The number of women self-weighing on most days per week (≥5 days/week on average) fluctuated over the study period ranging from 20 (83.3% of 24) in week 4 to 14 (58.3%) in week 24. **Fig 2** shows weekly patterns of DAT over the study period. Physical activity tracking remained relatively stable from 5.09 ± 2.39 days of tracking in week 1 to 4.00 ± 2.83 in week 24 (p = 0.34). Participants who self-monitored their weight 5 or more days per week on average achieved greater weight loss over the 6-month study period than those who weighed less than 5 days per week (-3.93 ± 5.65% vs. 0.19 ± 2.39%; *p* = 0.01) (**Fig 3**). Women who tracked their activity on 5 or more days per week lost 2.02 ± 2.83% of their baseline weight compared with 1.58 ± 3.92% among those who tracked activity less often; however, this difference was not statistically significant.

Nonadherence to DSW was associated with weight fluctuations (**Table 2**). For every 1-day of nonadherence to DSW, weight increased by 0.031 kg (95% CI: 0.012, 0.050; *p*<0.001). Adjusting for baseline weight did not alter our results (*β* = 0.031 kg, 95% CI: 0.012, 0.051; *p*<0.01). We did not find an association between nonadherence to DAT and changes in weight (*β* = -0.002 kg, 95% CI: -0.021, 0.017; *p* = 0.85). During periods of DSW and DAT (intercept terms), weight change was -0.028 kg (95% CI: -0.042, -0.014) and -0.017 (95% CI: -0.030, -0.004), respectively. Further adjustment for key demographic and clinical factors including age, education level, income level, marital status, menopause status, and cancer stage did not influence our results [DSW *β* = 0.0335 (95% CI: 0.023, 0.44) p<0.001; DAT *β =* 0.000 (95% CI: -0.023, 0.023) p = 0.996]. To examine the impact of DSW on the association between DAT and weight change in the INT+ group, we estimated the GEE with both DSW and DAT included in the model and found the association was slightly attenuated. However, these estimates did not change the interpretation of our findings (β = -0.001 kg, 95% CI: -0.027, 0.025; p = 0.94).

**Table 2:** Generalized estimating equation (GEE) models predicting weight change (kg) in relation to nonadherence to DSW and DAT^a^.

	*β* (95% CI)	p-value
*Self-weighing^b^*		
Intercept	-0.028 (-0.042, -0.014)	**<0.001**
Nonadherence to DSW	0.031 (0.012, 0.050)	**<0.01**
*Physical activity tracking^c^*		
Intercept	-0.017 (-0.030, -0.004)	**0.01**
Nonadherence to DAT	-0.002 (-0.021, 0.017)	0.85

^a^Nonadherence to DSW defined as one or more days without a weight measurement (from wireless scales). Nonadherence to DAT was defined as one or more days without activity tracking (<1000 total steps/day).

^b^Model for self-weighing reflects 2,858 weight measures.

^c^Model for physical activity tracking reflects 1,341 bouts of physical activity.

## Discussion

In this secondary analysis of a weight gain prevention trial among African American breast cancer survivors, we found that nonadherence to DSW was associated with weight increases. Specifically, for each 1-day of nonadherence to DSW, women gained, on average, 0.031 kg. Similarly, our results suggest that during periods of DSW and DAT, women lost weight. Further, we also observed differences in weight change among women who self-weighed at least five days per week compared to less than five days per week.

To our knowledge, this is one of the few studies to examine weight fluctuations in relation to DSW and DAT, and the first among breast cancer survivors. Our results are consistent with findings from two previous studies. One of the first studies to investigate temporal associations between nonadherence to DSW and weight changes was a secondary analysis of an 8-week worksite health promotion program with a one-year follow-up period by Herlander et al. (2014) [23]. Similar to our results, this study found that weight increased as days between two consecutive weight measurements became longer (β = 0.02, p<0.001) and weight decreased during periods of DSW (β = -0.12, p<0.001). Similarly, Pourzanjani et al. (2016) demonstrated that individuals who tracked their physical activity more frequently lost weight among a subset users of a commercial reward platform for aggregating healthy activities [22].

During periods of adherence to DSW and DAT, we found that weight decreased. It is likely that women who were weighing daily were also physically active. To explore this, we assessed the correlation between total days weighed and total days of physical activity tracking among the INT+ group, which were highly correlated (ρ = 0.87, *p*<0.001). Additional studies are warranted to disentangle the effects of DSW and DAT on weight fluctuations.

We also found that weight decreased by nearly 4% among women weighing at least 5 days per week, while weight increased 0.2% for those weighing less than 5 days, over the 6-month study period. These results are similar to the previous findings from weight control interventions examining self-weighing frequency and weight changes, though our study promoted weight gain prevention with small daily dietary behavior changes [7, 11–14, 26]. A previous study conducted a secondary analysis of self-weighing data to identify patterns of self-weighers: (1) high/consistent, who weighed more than 6 days per week regularly; (2) moderate/declined, who declined from 4–5 to 2 days of weighing per week; and (3) minimal/declined, who declined from 5–6 to 0 days of weighing per week. The high/consistent group had greater weight loss than the moderate/declined and minimal/declined groups at 6 (-10.2% vs. -5.5% vs. -2.0%) and 12 months (-9.9% vs. -5.6% vs. 0.7%) [12].

DSW and DAT are promising for weight gain prevention among high-risk groups, particularly African American breast cancer survivors. To our knowledge, this is the first study to examine DSW and DAT among breast cancer survivors. Further, we are the first to encourage self-monitoring behaviors in breast cancer survivors. As a result, acceptability and feasibility of DSW and DAT among breast cancer survivors remains unknown. We are currently analyzing qualitative data of interviews about DSW and DAT, but this work is outside the scope of this manuscript. Overall, the findings of our study have important implications for weight control among African American breast cancer survivors; however, more research is warranted to better understand the acceptability of DSW and DAT in breast cancer survivors and further evaluate interventions that encourage this approach. Inclusion of these tools in weight control interventions among populations vulnerable to weight gain can help interventions detect, in real-time, early signs of nonadherence and, importantly, weight gain.

### Limitations

Although our findings suggest that nonadherence to DSW and DAT is associated with weight gain, there are several limitations worth mentioning. Our study sample only included women who had regular access to the internet or computer and breast cancer survivors who were motivated to participate in our weight gain prevention study. Therefore, our findings may not be generalizable to breast cancer survivors with less access to technology or who were not as healthy or motivated. Although participants were strongly encouraged to wear activity trackers daily, it is possible that participants were physically active but did not wear their activity trackers. However, 72% of women in the INT+ group wore their activity trackers 5 or more days per week and the median total days worn was 162 out of 168 (96.4% of prescribed days).[17] Further, we are not able to determine whether the associations observed were due to the participants being adherent to the multicomponent intervention or that DSW and DAT actually led to changes in weight; however, we can conclude that regular self-weighing and activity tracking are associated with lower risk of weight gain among a sample of African American breast cancer survivors.

## Conclusions

Despite the noted limitations, the findings from this analysis of African American breast cancer survivors participating in a technology-based intervention study suggests that during periods of nonadherence to DSW, the risk of weight gain increases. Further, weight loss is associated with periods of DSW and DAT. To our knowledge, this is the first study to examine this among breast cancer survivors who are at risk of weight gain. Additional research is warranted in larger trials among breast cancer survivors to confirm our resuls. The findings can be useful in the development of future weight gain prevention studies among this high-risk population.
